# NLRP3 Inflammasome Activates Endothelial-to-Mesenchymal Transition via Focal Adhesion Kinase Pathway in Bleomycin-Induced Pulmonary Fibrosis

**DOI:** 10.3390/ijms242115813

**Published:** 2023-10-31

**Authors:** Wei-Chih Chen, Wen-Kuang Yu, Vincent Yi-Fong Su, Han-Shui Hsu, Kuang-Yao Yang

**Affiliations:** 1 Institute of Emergency and Critical Care Medicine, College of Medicine, National Yang Ming Chiao Tung University, Taipei 112, Taiwan; wiji.chen@gmail.com (W.-C.C.); hsuhs@vghtpe.gov.tw (H.-S.H.); 2 School of Medicine, College of Medicine, National Yang Ming Chiao Tung University, Taipei 112, Taiwan; wkyu2@vghtpe.gov.tw (W.-K.Y.); bsbipoke@hotmail.com (V.Y.-F.S.); 3 Department of Chest Medicine, Taipei Veterans General Hospital, Taipei 112, Taiwan; 4 Institute of Physiology, College of Medicine, National Yang-Ming University, Taipei 112, Taiwan; 5 Department of Internal Medicine, Taipei City Hospital, Taipei 110, Taiwan; 6 Division of Thoracic Surgery, Department of Surgery, Taipei Veterans General Hospital, Taipei 112, Taiwan; 7 Cancer Progression Research Center, National Yang-Ming University, Taipei 112, Taiwan

**Keywords:** nucleotide-binding domain leucine-rich repeat-containing receptor, pyrin domain-containing-3 (NLRP3) inflammasome, endothelial-to-mesenchymal transition, focal adhesion kinase, caspase-1 inhibitor, pulmonary fibrosis

## Abstract

Idiopathic pulmonary fibrosis has poor clinical outcomes despite antifibrotic treatment. The nucleotide-binding domain leucine-rich repeat-containing receptor, pyrin domain-containing-3 (NLRP3) inflammasome and endothelial-to-mesenchymal transition (EndoMT) were shown to be involved in the pathogenesis of pulmonary fibrosis. However, the detailed mechanism is unknown. Our study aimed to investigate the role of the NLRP3 inflammasome in the regulation of EndoMT in pulmonary fibrosis. The inhibition of the NLRP3 inflammasome via a caspase-1 inhibitor, Ac-YVAD-cmk (YVAD), was intraperitoneally administered to male C57BL/6 mice (8–12 weeks old) one hour before bleomycin intratracheal injection (1.5 U/kg). Immunohistochemical staining, Masson’s trichrome staining, enzyme-linked immunosorbent assay, immunofluorescence, and Western blotting were used to assess the activity of the NLRP3 inflammasome and EndoMT in lung samples from mice. Human pulmonary microvascular endothelial cells (HPMECs) were used as a model of EndoMT in vitro with YVAD and bleomycin stimulation. We observed the activation of the NLRP3 inflammasome and EndoMT (decreased vascular endothelial cadherin with increased alpha-smooth muscle actin and vimentin) in the lung samples after bleomycin. However, inhibition of the NLRP3 inflammasome significantly reduces EndoMT via inhibiting focal adhesion kinase (FAK). In vitro studies also confirmed these findings. In conclusion, NLRP3 inflammasome inhibition could reduce lung inflammation and fibrosis via the regulation of EndoMT by the FAK pathway.

## 1. Introduction

Lung fibrosis is commonly observed in the late stage of various pulmonary diseases, including interstitial lung disease (ILD) and acute respiratory distress syndrome (ARDS) [1,2]. Histopathological features of pulmonary fibrosis include abnormal collagen deposition, rapid increase in mesenchymal cells, and damage to lung architecture [3]. The median survival of idiopathic pulmonary fibrosis (IPF), the most-seen ILD, was less than 5 years [4]. Our previous work showed that both the early use of nintedanib or induced pluripotent stem cells might reduce lung inflammation and fibrosis in the murine bleomycin model [5,6]. It is thus important to give early treatment to reduce lung fibrosis.

Inflammasomes are activated during stress or infectious agents, enhancing the maturation of proinflammatory cytokines to participate in innate immunity [7]. One of them, known as the nucleotide-binding domain leucine-rich repeat-containing receptor, pyrin domain-containing-3 (NLRP3) inflammasome, is involved in neutrophil influx and elevated interleukin-1 beta (IL-1β) levels in bleomycin model; these effects are attenuated when these mice are treated with Z-YVAD-FMK, a caspase-1 inhibitor [8]. It has been reported that in diabetic wounds, multiple inflammatory cytokines, including IL-1β and TNF-α, are upregulated by activated NLRP3 inflammasomes [9,10]. The NLRP3 inflammasome is also activated in patients with IPF via extracellular adenosine triphosphate [11]. In addition, the NLRP3 inflammasome participates in the regulation of epithelial-to-mesenchymal transition (EMT) in fibrosis [12].

As observed in a pulmonary fibrosis model, fibroblasts could originate from endothelial-to-mesenchymal transition (EndoMT) [13]. The inhibition of EndoMT has been found to ameliorate pulmonary fibrosis in both lipopolysaccharide-induced lung injury and transforming growth factor-beta (TGF-β)-induced pulmonary fibrosis [14,15]. In addition, NLRP3 is involved in EndoMT in a mechanical stretch model [16].

In endothelial cells, focal adhesion kinase (FAK) is an important mediator of endothelial cytoskeletal interactions [17]. In addition, both increased FAK expression [18] and Tyr-397 phosphorylation of FAK [19] are found in association with the downregulation of vascular endothelial cadherin (VE-cadherin) and increased permeability in endothelial cells treated with hydrogen peroxide. Phosphorylation of FAK in Tyr397 was also found to regulate both EMT [20] and EndoMT [21]. Interestingly, the FAK signaling pathway was involved in the activation of the NLRP3 inflammasome in intestinal epithelial cells as well [22]. Activation of caspases was known to cleave FAK and cause cytoskeleton rearrangement in cell apoptosis [23].

Considering these facts, we hypothesized that the NLRP3 inflammasome has significant effects on EndoMT in bleomycin-induced pulmonary fibrosis, which could be attenuated by the inhibition of the NLRP3 inflammasome via a reduction in FAK expression. The aim of this study is to investigate the mechanisms of the NLRP3 inflammasome and EndoMT in bleomycin-induced pulmonary fibrosis.

## 2. Results

### 2.1. Inhibition of the NLRP3 Inflammasome Alleviated Bleomycin-Induced Pulmonary Fibrosis

**Figure 1:**
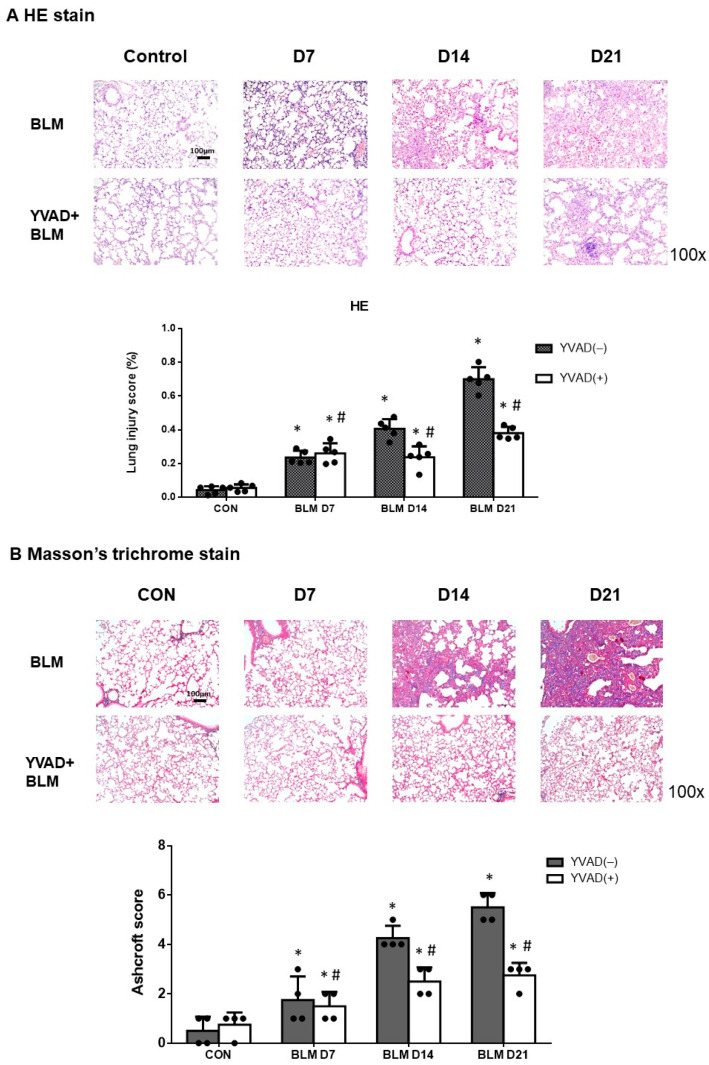
Inhibition of NLRP3 inflammasome improved histological features and reduced pulmonary fibrosis in mice with BLM-induced pulmonary fibrosis. (**A**) H&E stain and lung injury score demonstrated that lung inflammation and fibrosis were reduced in mice receiving Ac-YVAD-cmk (YVAD), a caspase-1 inhibitor to inhibit NLRP3 inflammasome signaling pathway compared with the bleomycin group; (**B**) Masson’s trichrome staining and the Ashcroft score showed that pulmonary fibrosis mice that received YVAD had significantly less pulmonary fibrosis compared with bleomycin group. * *p* < 0.05 compared with control, # *p* < 0.05 compared with BLM group. n = 5 per group.

The intratracheal administration of bleomycin-induced lung injury is evidenced histopathologically by the presence of alveolar infiltrates of neutrophils (Supplementary Figure S1), thickening of the alveolar walls and interstitium, and extensive fibrosis (Figure 1A). To investigate the role of NLRP3 inflammasomes in bleomycin-induced pulmonary fibrosis, we compared the lung sections from pulmonary fibrosis mice that underwent preventive treatment with Ac-YVAD-cmk (YVAD), a caspase-1 inhibitor to inhibit the NLRP3 inflammasome signaling pathway via intraperitoneal injection. Histological evaluation of the lungs seven days after bleomycin-induced pulmonary fibrosis showed that lung inflammation and fibrosis were significantly reduced by YVAD preventive treatment. Mice treated with YVAD had significantly decreased lung injury scores (Figure 1A). Masson’s trichrome staining of lung tissue and the Ashcroft score showed progressive pulmonary fibrosis seven days after bleomycin injection (Figure 1B). In contrast, preventive treatment with YVAD significantly reduced bleomycin-induced pulmonary fibrosis, both according to Masson’s trichrome staining and the Ashcroft score (Figure 1B).

### 2.2. Activation of NLRP3 Inflammasome in Bleomycin-Induced Pulmonary Fibrosis

**Figure 2:**
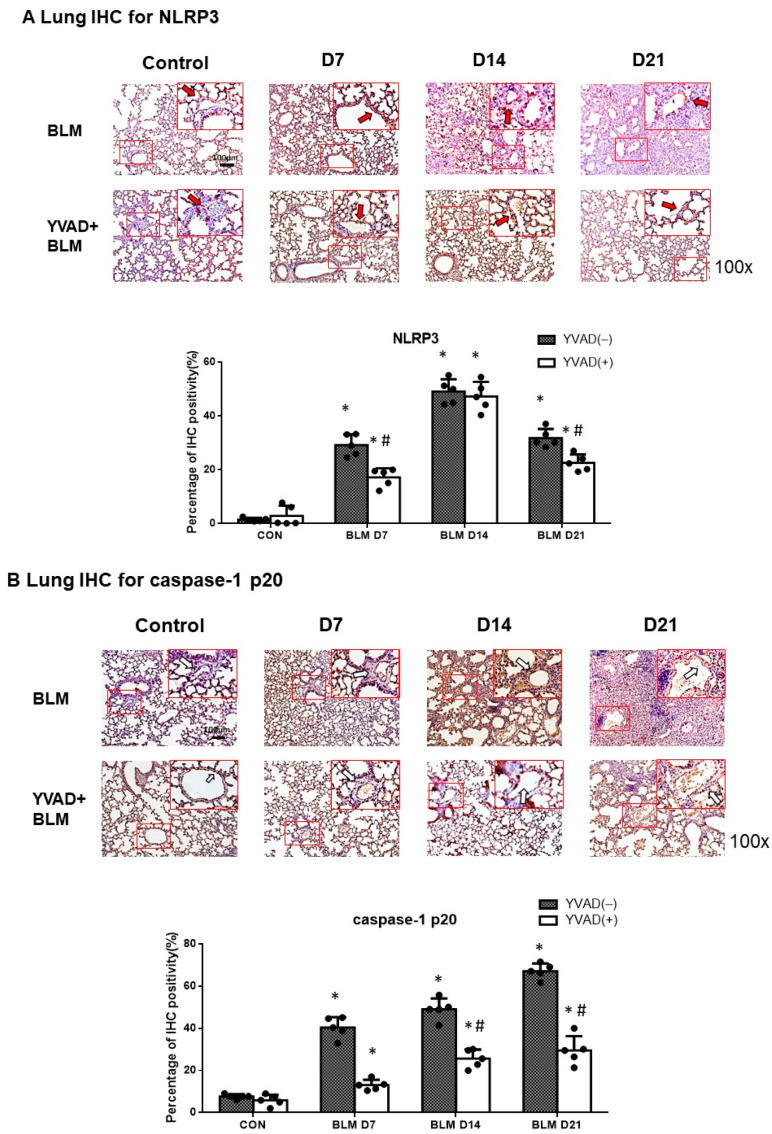

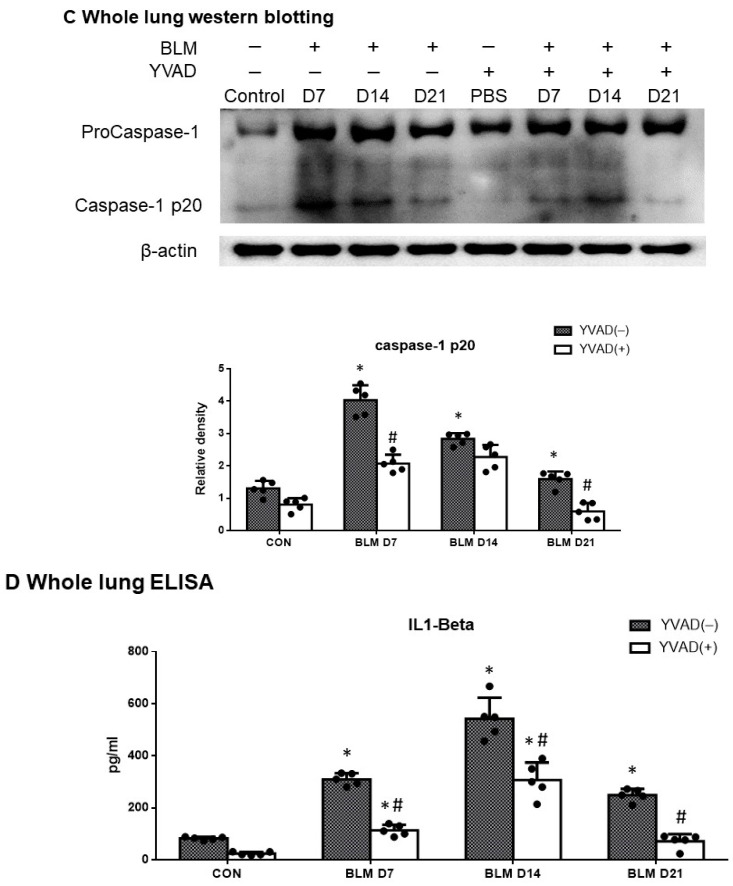
Activation of NLRP3 inflammasome in mice with BLM-induced pulmonary fibrosis. (**A**) IHC analysis showed that the administration of bleomycin increased NLRP3 inflammasome expression in lung tissue seven days after bleomycin injection, but YVAD decreased NLRP3 expression. Areas in red boxes were shown enlarged (arrows: pulmonary vascular endothelial cells); (**B**) IHC analysis of caspase-1 p20 as markers of NLRP3 inflammasome expression showed that the administration of bleomycin increased NLRP3 inflammasome expression in lung tissue seven days after bleomycin injection, but YVAD decreased caspase-1 p20 expression. Areas in red boxes were shown enlarged (arrows: pulmonary vascular endothelial cells); (**C**) Western blot assays of the whole lung extracts demonstrated increased caspase-1 p20 expression seven days after bleomycin administration, but YVAD attenuated caspase-1 p20 expression; (**D**) ELISA of the whole lung extracts showed that IL-1β levels increased significantly seven days after bleomycin administration. The treatment of YVAD attenuated IL-1β increased levels seven days after bleomycin administration. * *p* < 0.05 compared with control, # *p* < 0.05 compared with BLM group. n = 5 per group.

Immunohistochemistry (IHC) staining of the lung tissues showed that the expression levels of the NLRP3 inflammasome and caspase-1 p20 were significantly higher in the pulmonary fibrosis model group compared to the control group seven days after bleomycin injection (Figure 2A,B). Western blots of the whole lung extracts showed that the caspase-1 p20 subunit level was significantly higher in the pulmonary fibrosis model group compared to the control group seven days after bleomycin injection (Figure 2C). The enzyme-linked immunosorbent assay (ELISA) of the whole lung extracts showed that IL-1β levels were significantly higher in the pulmonary fibrosis model group compared to the control group seven days after bleomycin injection (Figure 2D).

Mice treated with YVAD one hour before bleomycin injection had significantly lower NLRP3 inflammasome and caspase-1 p20 subunit expression seven days after bleomycin injection compared to the bleomycin group; the effect persisted for 21 days (Figure 2A–C). IL-1β levels were also significantly lower seven days after bleomycin injection in the YVAD group compared to the bleomycin group; the effect persisted for 21 days (Figure 2D).

### 2.3. NLRP3 Inflammasome-Regulated Endothelial and Mesenchymal Markers in Bleomycin-Induced Pulmonary Fibrosis

**Figure 3:**
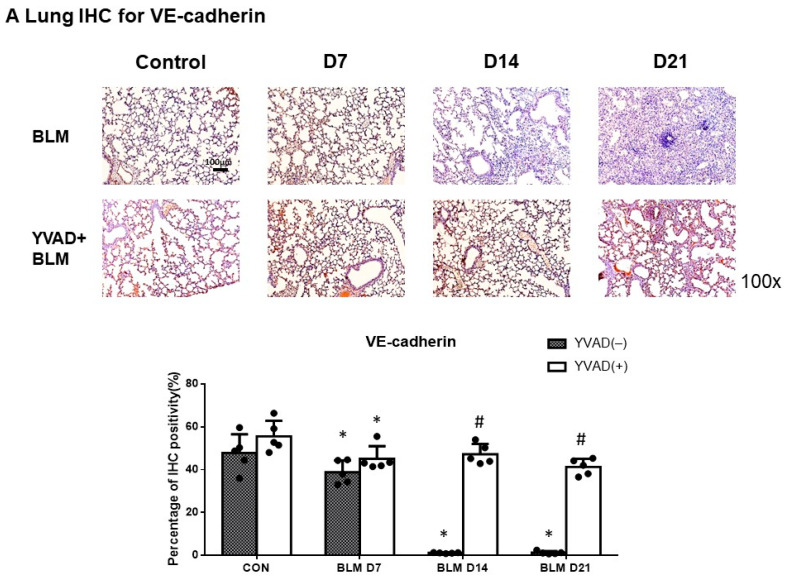

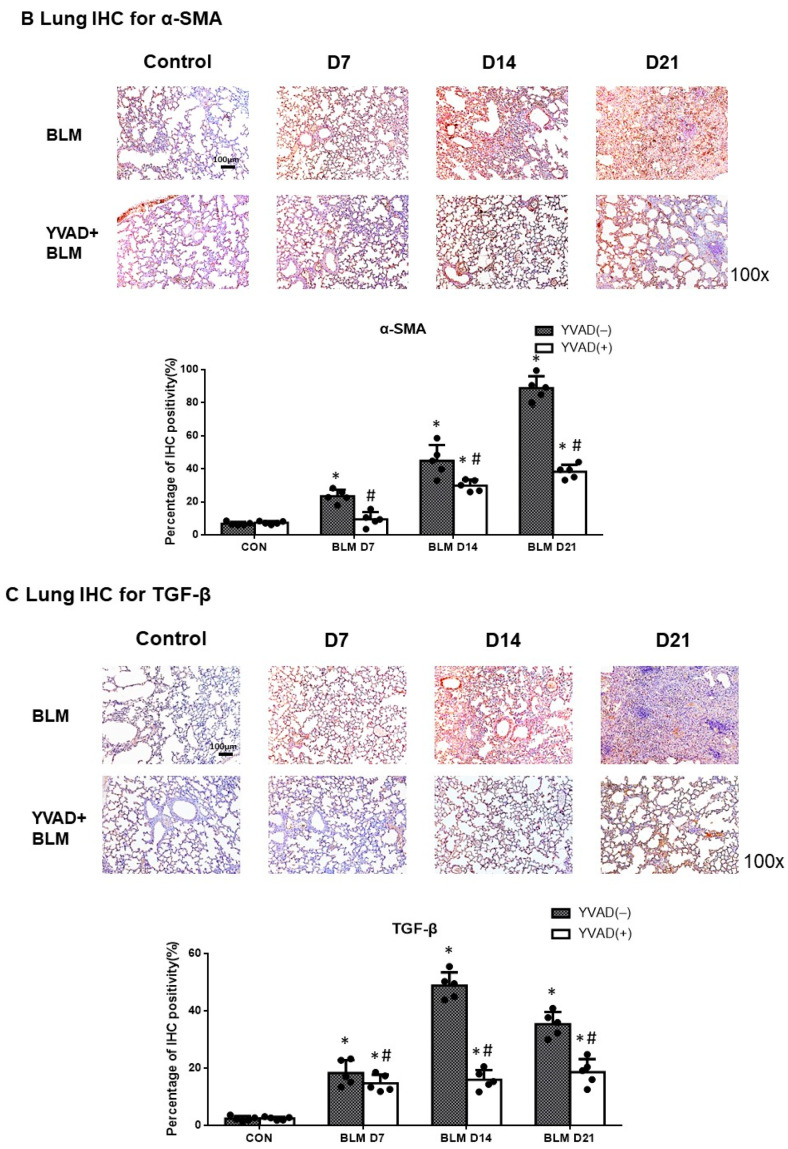

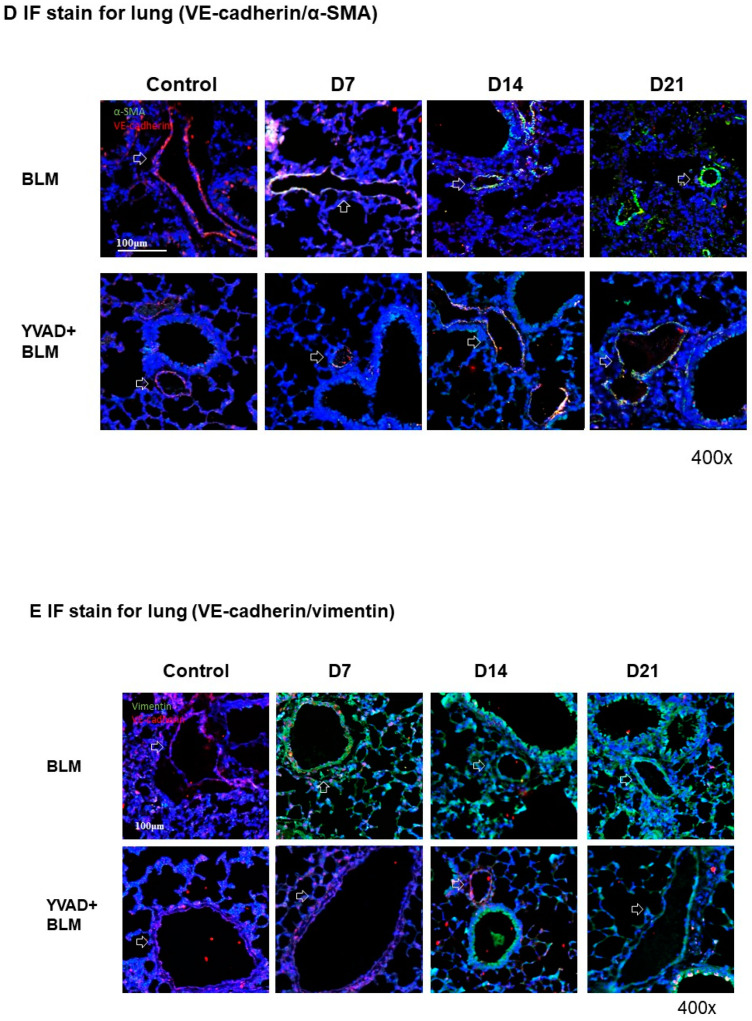

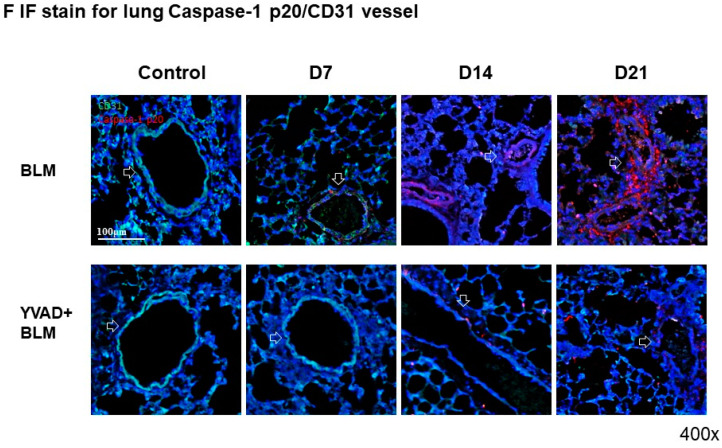
Inhibition of NLRP3 inflammasome diminished the process of endothelial to mesenchymal transition in BLM-induced pulmonary fibrosis. (**A**) IHC stain demonstrated that the levels of VE-cadherin significantly decreased seven days after BLM injection. Inhibition of NLRP3 inflammasome by YVAD significantly rescued VE-cadherin expression fourteen days after bleomycin injection; (**B**) IHC stain demonstrated levels of α-SMA increased significantly in the bleomycin group. Administration of YVAD decreased α-SMA levels seven days after bleomycin; (**C**) likewise, TGF-β levels increased significantly after bleomycin. Treatment of YVAD decreased TGF-β expression seven days after bleomycin; (**D**) immunofluorescence (IF) double staining of lung tissue demonstrated decreased expression of VE-cadherin with concomitant increased expression of α-SMA among pulmonary vascular endothelial cells seven days after bleomycin-induced pulmonary fibrosis. YVAD significantly preserved expression of VE-cadherin with decreased expression of α-SMA after bleomycin injection (arrows: pulmonary vascular endothelial cells); (**E**) IF double staining of lung tissue demonstrated decreased expression of VE-cadherin with concomitant increased expression of vimentin among pulmonary vascular endothelial cells seven days after bleomycin-induced pulmonary fibrosis. YVAD significantly preserved expression of VE-cadherin with decreased expression of vimentin after bleomycin injection (arrows: pulmonary vascular endothelial cells); (**F**) IF double staining of lung tissue demonstrated decreased expression of CD31 with concomitant increased expression of caspase-1 p20 among pulmonary vascular endothelial cells seven days after bleomycin injection. YVAD significantly preserved the expression of CD31 with decreased levels of caspase-1 p20 after bleomycin (arrows: pulmonary vascular endothelial cells). * *p* < 0.05 compared with control, # *p* < 0.05 compared with BLM group. n = 5 per group.

**Figure 4:**
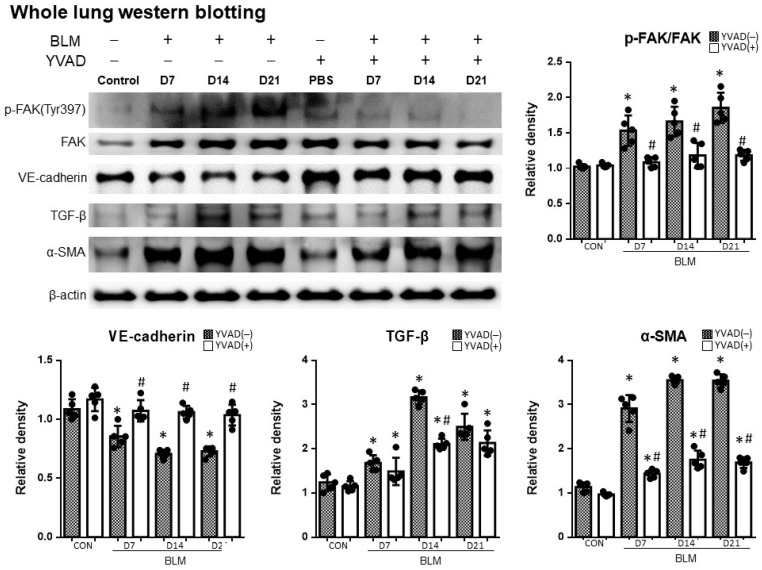
Inhibition of NLRP3 inflammasome reversed changes in protein expression in BLM-induced pulmonary fibrosis. Western blot assays of the whole lung extracts revealed increased p-FAK-to-FAK ratio, decreased VE-cadherin expression, and increased levels of TGF-β and α-SMA in bleomycin-induced pulmonary fibrosis. Inhibition of NLRP3 inflammasome by YVAD attenuated the p-FAK-to-FAK ratio while preserving VE-cadherin in bleomycin-induced pulmonary fibrosis. Additionally, YVAD reduced the expression of TGF-β and α-SMA. * *p* < 0.05 compared with control, # *p* < 0.05 compared with BLM group. n = 5 per group.

IHC staining of lung tissue and Western blots of the whole lung was performed to detect changes in endothelial and mesenchymal markers in lung tissue following the administration of bleomycin. A decrease in VE-cadherin expression levels was observed seven days after bleomycin use. Increases in TGF-β and α-SMA levels were noted seven days after bleomycin injection. In addition, the inhibition of the NLRP3 inflammasome via YVAD preventive treatment preserved VE-cadherin levels significantly after bleomycin-induced pulmonary fibrosis. The expression of TGF-β and α-SMA was reduced in YVAD-treated mice seven days after bleomycin-induced pulmonary fibrosis (Figure 3A–C and Figure 4). Immunofluorescence double staining was used to detect EndoMT in vessels in lung tissue after bleomycin injection. Decreases in VE-cadherin with concomitant increases of α-SMA expression levels among pulmonary vascular endothelial cells were observed seven days after bleomycin injection (Figure 3D). Similarly, decreases in VE-cadherin with concomitant increases in vimentin expression levels among pulmonary vascular endothelial cells were observed seven days after bleomycin injection (Figure 3E). Decreases in CD31 with concomitant increases of caspase-1 p20 among pulmonary vascular endothelial cells were also observed seven days after bleomycin injection (Figure 3F). Moreover, YVAD treatment significantly preserved VE-cadherin levels with decreased levels of vimentin and α-SMA in vascular endothelial cells after bleomycin-induced pulmonary fibrosis (Figure 3D,E). YVAD also significantly preserved CD31 levels with decreased expression of caspase-1 p20 in vascular endothelial cells after bleomycin use (Figure 3F).

### 2.4. Inhibition of the NLRP3 Inflammasome Reduced FAK Activity in Bleomycin-Induced Pulmonary Fibrosis

Western blots of the whole lung were applied to detect p-FAK changes in the lung tissue after the administration of bleomycin. The p-FAK-to-FAK ratio significantly increased after bleomycin administration. In contrast, the inhibition of the NLRP3 inflammasome via the administration of YVAD attenuated the increase in the p-FAK-to-FAK ratio in bleomycin-induced pulmonary fibrosis (Figure 4).

### 2.5. Inhibition of the NLRP3 Inflammasome Reduced Bleomycin-Induced EndoMT In Vitro

**Figure 5:**
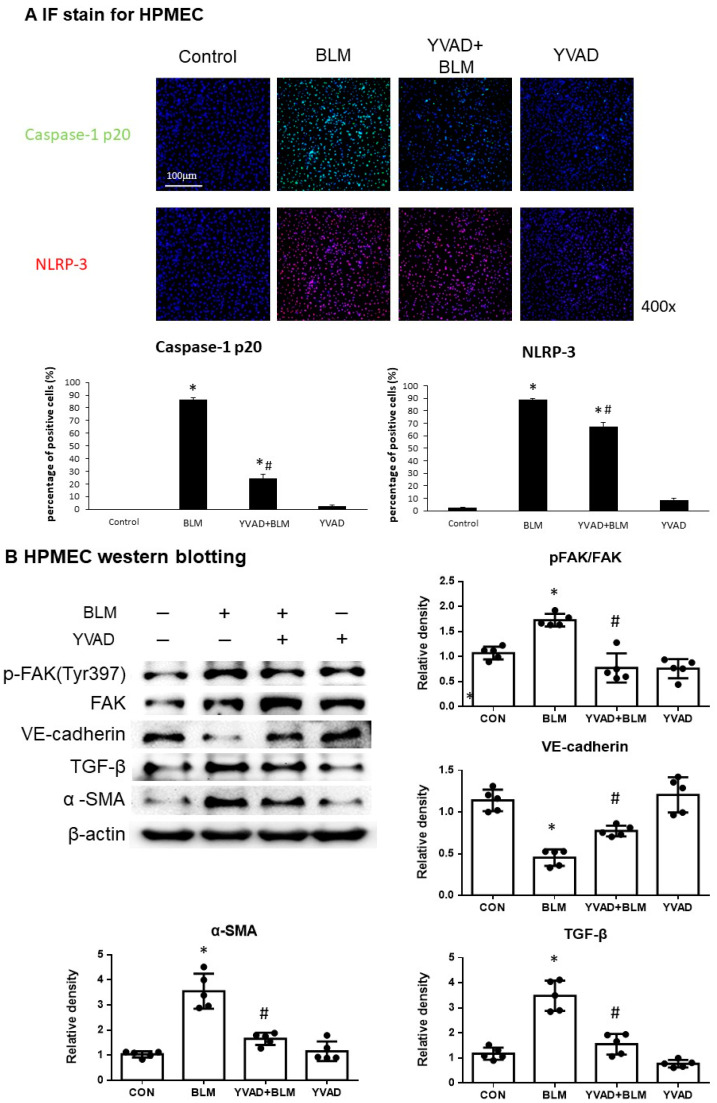

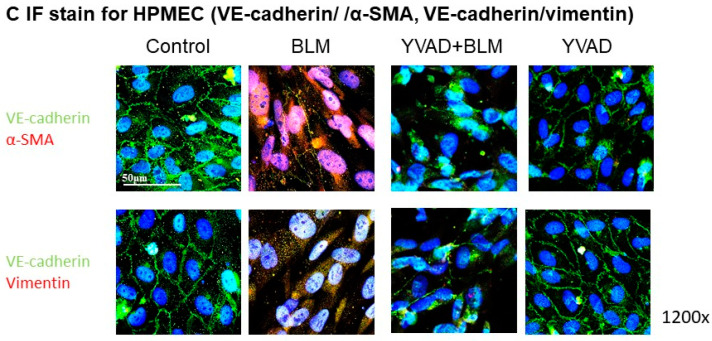
Inhibition of NLRP3 inflammasome reduced BLM-induced EndoMT in vitro. (**A**) IF stain of HPMEC showed that the expression of caspase-1 p20 and NLRP3 was increased significantly in HPMEC following the administration of bleomycin. However, inhibition of NLRP3 inflammasome by YVAD reduced the increases of caspase-1 p20 and NLRP3 expression after bleomycin use; (**B**) Western blot assays of the whole cell lysates of HPMEC revealed increased p-FAK-to-FAK ratio, expressive levels of TGF-β and α-SMA after BLM stimulation, and decreased VE-cadherin expression. Administration of YVAD diminished the increased levels of p-FAK-to-FAK ratio, TGF-β, and α-SMA expression after bleomycin injection. Moreover, YVAD significantly recovered the decreased levels of VE-cadherin after bleomycin stimulation; (**C**) IF double staining of HPMEC demonstrated decreased expression of VE-cadherin with concomitant increased expression of α-SMA after bleomycin stimulation. Similarly, decreases in VE-cadherin with concomitant increases in vimentin expression levels were observed in HPMEC after bleomycin administration. YVAD significantly preserved VE-cadherin levels with decreased expression of α-SMA and vimentin after bleomycin use. * *p* < 0.05 compared with control, # *p* < 0.05 compared with BLM group.

The immunofluorescence stain of human pulmonary microvascular endothelial cells (HPMECs) revealed that the expression of caspase-1 p20 and NLRP3 was significantly increased in HPMEC following the stimulation of bleomycin. However, the inhibition of the NLRP3 inflammasome by YVAD reduced the increases of caspase-1 p20 and NLRP3 after bleomycin stimulation (Figure 5A). Western blots of HPMEC were performed to detect various protein changes in HPMEC following the administration of bleomycin. The p-FAK-to-FAK ratio and TGF-β and α-SMA levels significantly increased after bleomycin, while VE-cadherin levels decreased; however, the inhibition of the NLRP3 inflammasome by YVAD reduced the increase in p-FAK-to-FAK ratio, TGF-β and α-SMA levels, and increased the VE-cadherin levels after bleomycin use (Figure 5B).

Immunofluorescence double staining of HPMEC was performed to detect EndoMT in HPMEC following the stimulation of bleomycin. Decreases in VE-cadherin with concomitant increases in α-SMA expression levels were observed. Similarly, decreases in VE-cadherin with concomitant increases in vimentin expression levels were also observed. However, the inhibition of the NLRP3 inflammasome by YVAD significantly preserved VE-cadherin expression levels with decreased expression of α-SMA and vimentin after bleomycin stimulation (Figure 5C).

## 3. Discussion

In the present study, the role of the NLRP3 inflammasome in the regulation of EndoMT in pulmonary fibrosis was demonstrated. In addition to the inhibition of NLRP3 inflammasome signaling pathways, the caspase-1 inhibitor had an antifibrotic effect by suppressing the EndoMT by reducing the expression of FAK.

The over-deposition of extracellular matrix synthesized by myofibroblasts is the key feature observed in lung tissue with fibrosis [3]. EndoMT was confirmed in Tie2-Cre/CAG-CAT-LacZ double transgenic mice with bleomycin administration to induce pulmonary fibrosis by increasing β-galactosidase activity encoded by the LacZ gene [13]. Besides bleomycin, other pulmonary fibrosis models, including radiation and mechanical ventilation models, also demonstrated evidence of EndoMT [16,24]. In the lung tissues derived from patients with systemic sclerosis-associated interstitial lung disease, EndoMT was observed with cells expressing endothelial and mesenchymal cell-specific markers concomitantly [25]. In a rat model, an NF-κB-dependent Jagged1/Notch1 signaling pathway was activated, and α-SMA expression was increased in pulmonary microvascular endothelial cells after bleomycin use [26]. However, evidence of EndoMT in patients with IPF was not observed. Our study result was also consistent with the result of the previously published data by confirming the presence of EndoMT in bleomycin-induced pulmonary fibrosis.

In preclinical experimental studies, several interventions have been proven to attenuate EndoMT and lung inflammation and fibrosis. A dipeptidyl peptidase 4 inhibitor, vildagliptin, could successfully downregulate EndoMT and reduce lung fibrosis in lipopolysaccharides-induced lung injury [14]. The overexpression of C-X-C chemokine receptor type 7 (CXCR7) attenuates EndoMT in TGF-β-induced pulmonary fibrosis via a feedback mechanism [15]. Of note, a study also discovered the role of NLRP3 inflammasome activation in EndoMT and lung fibrosis caused by mechanical ventilation [16]. NLRP3 knockout mice further ameliorated the activity of EndoMT compared to wild-type ones. Our study further found that YVAD, a caspase-1 inhibitor, could effectively alleviate bleomycin-induced pulmonary fibrosis both by downregulating the NLRP3 inflammasome and reducing the activity of EndoMT. FAK is known to help in cell adhesion to the extracellular matrix and is considered a central molecule for TGF-β-induced myofibroblast differentiation [27,28]. The inhibition of FAK could suppress fibrogenic responses by blocking TGF-β signaling. In human intestine cells, the activation of the NLRP3 inflammasome via FAK-mediated integrin signaling upon exposure to bacterial toxin is observed [29]. FAK activation is also required for EndoMT in tumorigenesis related to Kaposi’s sarcoma herpesvirus [30]. In our study, we demonstrated that YVAD could effectively decrease the p-FAK-to-FAK ratio, subsequently reducing the decrease in VE-cadherin and downregulating the process of EndoMT.

Our study had several limitations. First, our results were based on the effects of YVAD on a murine bleomycin model and an in vitro human endothelial cell model. Hence, it is unclear whether caspase-1 inhibitor exhibits similar clinical benefits in patients with fibrosing lung diseases. Second, we administered YVAD before bleomycin, and the use of YVAD successfully attenuated lung inflammation and fibrosis that occurred later. Whether the different timings of YVAD administration will have similar effects requires further examination. Third, NLRP3 knockout mice were unavailable at our lab; thus, we used a caspase-1 inhibitor to design the experiments. Since NLRP3 is dispensable in various chronic inflammations [31], future study designs should consider using these knockout mice to carefully address the role of NLRP3 and caspase-1 in bleomycin-induced pulmonary fibrosis. The complete inactivation of the NLRP3 gene could better understand the NLRP3 inflammasome and caspase-1 in the pathogenesis of bleomycin-induced pulmonary fibrosis compared with pharmacological inhibition.

## 4. Materials and Methods

### 4.1. Experimental Animals

We purchased male C57BL/6 mice (8–12 weeks old with a weight of 20–25 g) from the National Experimental Animal Center (Taipei, Taiwan). They were kept in standard plastic animal cages with husk bedding at 25  ± 2 °C with a 12 h light/dark cycle. All of them were provided with food and water ad libitum and maintained under specific pathogen-free conditions. All experiments complied with the Institutional Animal Care and Use Committee-approved protocols (TVGH IACUC No. 2017-076, 30 June 2017).

### 4.2. Animal Model

The mice were intratracheally injected with bleomycin sulfate (BLM) (Merck, Darmstadt, Germany) at a dose of 1.5 U/kg in 50 μL phosphate-buffered saline (PBS) to induce pulmonary fibrosis in a protocol adapted from our previous experiments after anesthesia [5]. The control mice received 50 μL PBS by intratracheal injection. In designated experiments, one hour before bleomycin injection, C57BL/6 mice in the treatment group were intraperitoneally injected with 8 mg/kg of Ac-YVAD-cmk (YVAD) (InvivoGen, San Diego, CA, USA), a caspase-1 inhibitor [32]. Animals in each group were sacrificed on days 7, 14, and 21. C57BL/6 mice in the YVAD+PBS group were also intraperitoneally injected with 8 mg/kg of Ac-YVAD-cmk. The control group mice were examined at the same time point as the treated mice and pooled from the different time points. Five mice were used in each group.

### 4.3. Histology and Immunohistochemistry

Lung tissues were fixed in 4% paraformaldehyde for 10 min, embedded in paraffin, and cut into 4 µm thick sections. Staining for NLRP3 (1:100, ab214185, Abcam, Cambridge, UK), anti-caspase 1 (p20) (1:1000, AG-20B-0042-C100, AdipoGen AG, Liestal, Switzerland), vascular endothelial cadherin (VE-cadherin) (1:100, ab33168, Abcam, Cambridge, UK), and alpha-smooth muscle actin (α-SMA) (1:100, 14395-1-AP, Proteintech, Rosemont, IL, USA) was performed using the Envision ^®^ + Dual Link System-HRP (DAB+) kits (K4065, DAKO, Carpinteria, CA, USA). Briefly, the sections were deparaffinized with xylene, dehydrated with ethanol, and subsequently heated in 0.01 M citrate buffer (pH 6.0). Endogenous peroxidase activity was inhibited in 3% H_2_O_2_ for 10 min at room temperature, and the sections were blocked with a blocking buffer (K4065 kit). Secondary anti-rabbit, antibody-coated polymer peroxidase complexes (K4065 kit) were subsequently administered for 30 min at room temperature, followed by substrate/chromogen treatment (K4065 kit), and further incubation for 5–15 s at room temperature. The sections were counterstained with hematoxylin (109249, Merck, Darmstadt, Germany) for 10 s and subsequently washed in running water for 10 min. The sections were observed and pictured using an Olympus AX80 fluorescence microscope (Olympus America, Melville, NY, USA). The percentage of immunohistochemistry (IHC) signal per field was analyzed using an image process software (Image-Pro Plus, Version 5.1.0.20, Media Cybernetics, Inc., Silver Spring, MD, USA).

### 4.4. Lung Injury Score

By blinded method, two investigators independently evaluated each hematoxylin and eosin and IHC stained slide. To evaluate the severity of the pathology, 10 random fields were used on each slide at 100× magnification. Within each field, points were assigned according to the predetermined criteria [33].

### 4.5. Masson’s Trichrome Staining

Lung tissues were fixed in 4% paraformaldehyde for 10 min, embedded in paraffin, and cut into 4 µm thick sections. These sections were stained utilizing the Trichrome Stain Kit (#ab150686, Abcam, Cambridge, UK).

### 4.6. Ashcroft Scale

To quantify the severity of pulmonary fibrosis by histology, the Ashcroft scale was used. Within each field, the points were calculated according to the criteria [34].

### 4.7. ELISA (Enzyme-Linked Immunosorbent Assay)

Mouse lung tissues were homogenized in a lysis buffer with protease inhibitors. Cytokine levels were quantitated using mouse IL-1β (MLB00C, R&D Systems, Inc., Minneapolis, MN, USA). The minimum detectable dose was 0.46 pg/mL for mouse IL-1β.

### 4.8. Western Blotting

Mouse lung tissues were homogenized in a lysis buffer with protease inhibitors (1862209, Thermo, Waltham, MA, USA). Protein homogenate was resolved equally on 7.5–10% sodium dodecyl sulfate-polyacrylamide electrophoresis gels and transferred onto the polyvinylidene fluoride membranes. Blots were blocked in a tris-buffered saline with Tween (TBST) containing 5% milk and directly detected with the following primary antibodies: anti-caspase 1 (p20) (1:1000, AG-20B-0042-C100, AdipoGen AG, Liestal, Switzerland), FAK (1:1000, #3285, Cell Signaling Technology, Danvers, MA, USA), p-FAK (1:500, #3284, Cell Signaling Technology, Danvers, MA, USA), VE-cadherin (1:1000, ab33168, Abcam, Cambridge, UK), TGF-β (1:500, #3711, Cell Signaling TECHNOLOGY, Danvers, MA, USA), α-SMA (1:1500, 14395-1-AP, Proteintech, Rosemont, IL, USA), and β-actin (1:5000, 20536-1-AP, Proteintech, Rosemont, IL, USA). Blots were subsequently washed in TBST, incubated in horseradish peroxidase secondary antibodies, and probed using an enhanced chemiluminescence substrate (Pierce Biochemicals, ThermoFisher SCIENTIFIC, Waltham, MA, USA). Each blot was exposed to a film, and the relative density of immunoreactive bands was determined using an ImageJ program, Version 1.53q (National Institute of Health, Bethesda, MD, USA).

### 4.9. Immunofluorescence

The sections were deparaffinized with xylene, dehydrated with ethanol, and then heated in 0.01 M citrate buffer (pH 6.0). At room temperature, after blocked with 3% fetal bovine serum (FBS) (in PBS) for 60 min, VE-cadherin (1:100, ab33168, Abcam, Cambridge, UK), α-SMA (1:100, 14395-1-AP, Proteintech, Rosemont, IL, USA), vimentin (1:100, V6630, Sigma, St. Louis, MO, USA), CD31 (1:1000, ab28364, Abcam, Cambridge, UK), and caspase-1 p20 (1:1000, AF4005, Affinity Biosciences, Cincinnati, OH, USA) were used overnight at 4 °C. On the second day, goat anti-rabbit immunoglobulin G (IgG) H&L (Alexa Fluor^®^ 488, Thermo Fisher Scientific, Waltham, MA, USA) (1:400, ab150077, Abcam, Cambridge, UK) and goat anti-rabbit IgG H&L (Cy5 ^®^, Thermo Fisher Scientific, Waltham, MA, USA) (1:400, ab6564, Abcam, Cambridge, UK) as secondary antibodies were incubated at 37 °C for 2 h. The slides were mounted by mounting medium with 4′, 6-diamidino-2-phenylindole (DAPI) (H-1200, Vector Laboratories, Burlingame, CA, USA) to obtain nuclear staining. Cell images were taken on a confocal microscope (FV10i, Olympus America, Melville, NY, USA).

### 4.10. In Vitro Stimulation and Culture of Human Pulmonary Microvascular Endothelial Cells (HPMEC)

HPMECs were used as an in vitro model of EndoMT. The cells were obtained from ScienCell Company (Carlsbad, CA, USA) and grown in an endothelial cell medium (1001, including 1X Endothelial Cell Growth Supplement, 1X Penicillin/Streptomycin Solution, and 5% FBS). The incubation condition was in room air with 5% CO_2_ at 37 °C and 95% humidity, and cell cultures were expanded by performing brief trypsinization with 0.25% trypsin in PBS containing 0.025% ethylenediaminetetraacetic acid. The experiments were conducted on passage 2–3 HPMECs. These HPMECs were stimulated with YVAD (50 μM/mL) for 30 min and treated with bleomycin (100 mU/mL) for 90 min. The cell cultures were removed for all liquid and washed 2 times with 1× PBS. Then, cells were fixed with 4% paraformaldehyde. After permeabilized with 0.5% Triton X-100 (in PBS) and blocked with 3% FBS (in PBS) for 60 min at room temperature, NLRP3 (1:100, ab214185, Abcam, Cambridge, UK) and anti-caspase 1 (p20) (1:1000, AG-20B-0042-C100, AdipoGen AG, Liestal, Switzerland) as primary antibodies were used overnight at 4 °C. The second day, goat anti-rabbit IgG H&L (Alexa Fluor^®^ 488) (1:400, ab150077, Abcam, Cambridge, UK) and goat anti-rabbit IgG H&L (Cy5 ^®^) (1:400, ab6564, Abcam, Cambridge, UK) as secondary antibodies were incubated at 37 °C for 2 h. The culture slides were mounted by mounting medium with DAPI (H-1200, Vector Laboratories, Burlingame, CA, USA) to obtain nuclear staining. Cells images were taken on a confocal microscope (FV10i, Olympus America, Melville, NY, USA).

### 4.11. Statistics

The animals were prepared and studied simultaneously. Separate mice were used for the IHC, permeability assays, Western blotting, Ashcroft scale, ELISA, and immunofluorescence analyses. Data were presented as mean ± standard deviation or standard error of mean for each experimental group. One-way analysis of variance and the Tukey–Kramer multiple comparisons test or pair-wise Student’s *t*-test were applied. A *p* value less than 0.05 was considered statistically significant.

## 5. Conclusions

In conclusion, we identified the novel role of the NLRP3 inflammasome in the regulation of EndoMT in bleomycin-induced lung inflammation and fibrosis. This effect was reduced by the inhibition of the NLRP3 inflammasome and the FAK signaling pathways by YVAD. These findings provide further evidence that caspase-1 inhibitors may ameliorate pulmonary fibrosis by blocking NLRP3 inflammasome and downregulating EndoMT. The inhibition of NLRP3 inflammasome may be a potential target in the management of fibrosing lung diseases.

## Data Availability

The data presented in this study are available upon request from the corresponding author.

## Supplementary Materials

The following supporting information can be downloaded at: https://www.mdpi.com/article/10.3390/ijms242115813/s1.

## References

[1] Travis W.D., Costabel U., Hansell D.M., King T.E., Lynch D.A., Nicholson A.G., Ryerson C.J., Ryu J.H., Selman M., Wells A.U. (2013). An official American Thoracic Society/European Respiratory Society statement: Update of the international multidisciplinary classification of the idiopathic interstitial pneumonias. Am. J. Respir. Crit. Care Med..

[2] Ware L.B., Matthay M.A. (2000). The acute respiratory distress syndrome. N. Engl. J. Med..

[3] Wuyts W.A., Agostini C., Antoniou K.M., Bouros D., Chambers R.C., Cottin V., Egan J.J., Lambrecht B.N., Lories R., Parfrey H. (2013). The pathogenesis of pulmonary fibrosis: A moving target. Eur. Respir. J..

[4] Nathan S.D., Shlobin O.A., Weir N., Ahmad S., Kaldjob J.M., Battle E., Sheridan M.J., du Bois R.M. (2011). Long-term course and prognosis of idiopathic pulmonary fibrosis in the new millennium. Chest.

[5] How C.K., Chien Y., Yang K.Y., Shih H.C., Juan C.C., Yang Y.P., Chiou G.Y., Huang P.I., Chang Y.L., Chen L.K. (2013). Induced pluripotent stem cells mediate the release of interferon gamma-induced protein 10 and alleviate bleomycin-induced lung inflammation and fibrosis. Shock.

[6] Chen W.C., Chen N.J., Chen H.P., Yu W.K., Su V.Y., Chen H., Wu H.H., Yang K.Y. (2020). Nintedanib Reduces Neutrophil Chemotaxis via Activating GRK2 in Bleomycin-Induced Pulmonary Fibrosis. Int. J. Mol. Sci..

[7] Schroder K., Tschopp J. (2010). The inflammasomes. Cell.

[8] Gasse P., Riteau N., Charron S., Girre S., Fick L., Petrilli V., Tschopp J., Lagente V., Quesniaux V.F., Ryffel B. (2009). Uric acid is a danger signal activating NALP3 inflammasome in lung injury inflammation and fibrosis. Am. J. Respir. Crit. Care Med..

[9] Ding Y., Ding X., Zhang H., Li S., Yang P., Tan Q. (2022). Relevance of NLRP3 Inflammasome-Related Pathways in the Pathology of Diabetic Wound Healing and Possible Therapeutic Targets. Oxid. Med. Cell. Longev..

[10] Geng K., Ma X., Jiang Z., Huang W., Gao C., Pu Y., Luo L., Xu Y., Xu Y. (2021). Innate Immunity in Diabetic Wound Healing: Focus on the Mastermind Hidden in Chronic Inflammatory. Front. Pharmacol..

[11] Riteau N., Gasse P., Fauconnier L., Gombault A., Couegnat M., Fick L., Kanellopoulos J., Quesniaux V.F., Marchand-Adam S., Crestani B. (2010). Extracellular ATP is a danger signal activating P2X7 receptor in lung inflammation and fibrosis. Am. J. Respir. Crit. Care Med..

[12] Tian R., Zhu Y., Yao J., Meng X., Wang J., Xie H., Wang R. (2017). NLRP3 participates in the regulation of EMT in bleomycin-induced pulmonary fibrosis. Exp. Cell Res..

[13] Hashimoto N., Phan S.H., Imaizumi K., Matsuo M., Nakashima H., Kawabe T., Shimokata K., Hasegawa Y. (2010). Endothelial-mesenchymal transition in bleomycin-induced pulmonary fibrosis. Am. J. Respir. Cell Mol. Biol..

[14] Suzuki T., Tada Y., Gladson S., Nishimura R., Shimomura I., Karasawa S., Tatsumi K., West J. (2017). Vildagliptin ameliorates pulmonary fibrosis in lipopolysaccharide-induced lung injury by inhibiting endothelial-to-mesenchymal transition. Respir. Res..

[15] Guan S., Zhou J. (2017). CXCR7 attenuates the TGF-beta-induced endothelial-to-mesenchymal transition and pulmonary fibrosis. Mol. Biosyst..

[16] Lv Z., Wang Y., Liu Y.J., Mao Y.F., Dong W.W., Ding Z.N., Meng G.X., Jiang L., Zhu X.Y. (2018). NLRP3 Inflammasome Activation Contributes to Mechanical Stretch-Induced Endothelial-Mesenchymal Transition and Pulmonary Fibrosis. Crit. Care Med..

[17] Infusino G.A., Jacobson J.R. (2012). Endothelial FAK as a therapeutic target in disease. Microvasc. Res..

[18] Yang S., Yip R., Polena S., Sharma M., Rao S., Griciene P., Gintautas J., Jerome H. (2004). Reactive oxygen species increased focal adhesion kinase production in pulmonary microvascular endothelial cells. Proc. West. Pharmacol. Soc..

[19] Usatyuk P.V., Natarajan V. (2005). Regulation of reactive oxygen species-induced endothelial cell-cell and cell-matrix contacts by focal adhesion kinase and adherens junction proteins. Am. J. Physiol. Lung Cell. Mol. Physiol..

[20] Cano A., Perez-Moreno M.A., Rodrigo I., Locascio A., Blanco M.J., del Barrio M.G., Portillo F., Nieto M.A. (2000). The transcription factor snail controls epithelial-mesenchymal transitions by repressing E-cadherin expression. Nat. Cell Biol..

[21] Kokudo T., Suzuki Y., Yoshimatsu Y., Yamazaki T., Watabe T., Miyazono K. (2008). Snail is required for TGFbeta-induced endothelial-mesenchymal transition of embryonic stem cell-derived endothelial cells. J. Cell Sci..

[22] Thinwa J., Segovia J.A., Bose S., Dube P.H. (2014). Integrin-mediated first signal for inflammasome activation in intestinal epithelial cells. J. Immunol..

[23] Lu Q., Rounds S. (2012). Focal adhesion kinase and endothelial cell apoptosis. Microvasc. Res..

[24] Choi S.H., Hong Z.Y., Nam J.K., Lee H.J., Jang J., Yoo R.J., Lee Y.J., Lee C.Y., Kim K.H., Park S. (2015). A Hypoxia-Induced Vascular Endothelial-to-Mesenchymal Transition in Development of Radiation-Induced Pulmonary Fibrosis. Clin. Cancer. Res..

[25] Mendoza F.A., Piera-Velazquez S., Farber J.L., Feghali-Bostwick C., Jimenez S.A. (2016). Endothelial Cells Expressing Endothelial and Mesenchymal Cell Gene Products in Lung Tissue From Patients with Systemic Sclerosis-Associated Interstitial Lung Disease. Arthritis Rheumatol..

[26] Yin Q., Wang W., Cui G., Yan L., Zhang S. (2018). Potential role of the Jagged1/Notch1 signaling pathway in the endothelial-myofibroblast transition during BLM-induced pulmonary fibrosis. J. Cell. Physiol..

[27] Thannickal V.J., Lee D.Y., White E.S., Cui Z., Larios J.M., Chacon R., Horowitz J.C., Day R.M., Thomas P.E. (2003). Myofibroblast differentiation by transforming growth factor-beta1 is dependent on cell adhesion and integrin signaling via focal adhesion kinase. J. Biol. Chem..

[28] Liu S., Xu S.W., Kennedy L., Pala D., Chen Y., Eastwood M., Carter D.E., Black C.M., Abraham D.J., Leask A. (2007). FAK is required for TGFbeta-induced JNK phosphorylation in fibroblasts: Implications for acquisition of a matrix-remodeling phenotype. Mol. Biol. Cell.

[29] Chung I.C., OuYang C.N., Yuan S.N., Li H.P., Chen J.T., Shieh H.R., Chen Y.J., Ojcius D.M., Chu C.L., Yu J.S. (2016). Pyk2 activates the NLRP3 inflammasome by directly phosphorylating ASC and contributes to inflammasome-dependent peritonitis. Sci. Rep..

[30] Sharma-Walia N., Patel K., Chandran K., Marginean A., Bottero V., Kerur N., Paul A.G. (2012). COX-2/PGE2: Molecular ambassadors of Kaposi’s sarcoma-associated herpes virus oncoprotein-v-FLIP. Oncogenesis.

[31] Davis B.K., Wen H., Ting J.P. (2011). The inflammasome NLRs in immunity, inflammation, and associated diseases. Annu. Rev. Immunol..

[32] Van Opdenbosch N., Gurung P., Vande Walle L., Fossoul A., Kanneganti T.D., Lamkanfi M. (2014). Activation of the NLRP1b inflammasome independently of ASC-mediated caspase-1 autoproteolysis and speck formation. Nat. Commun..

[33] Matute-Bello G., Winn R.K., Jonas M., Chi E.Y., Martin T.R., Liles W.C. (2001). Fas (CD95) induces alveolar epithelial cell apoptosis in vivo: Implications for acute pulmonary inflammation. Am. J. Pathol..

[34] Hubner R.H., Gitter W., El Mokhtari N.E., Mathiak M., Both M., Bolte H., Freitag-Wolf S., Bewig B. (2008). Standardized quantification of pulmonary fibrosis in histological samples. BioTechniques.

