# Supplementary material for: NLRP3 Inflammasome Activates Endothelial-to-Mesenchymal Transition via Focal Adhesion Kinase Pathway in Bleomycin-Induced Pulmonary Fibrosis
Source: Int J Mol Sci. 2023 Oct 31;24(21):15813. doi: 10.3390/ijms242115813 (PMC10648980; doi:10.3390/ijms242115813)
Supplement: Supplementary file 1 [file ijms-24-15813-s001.zip › ijms-2643841-supplementary.pdf]

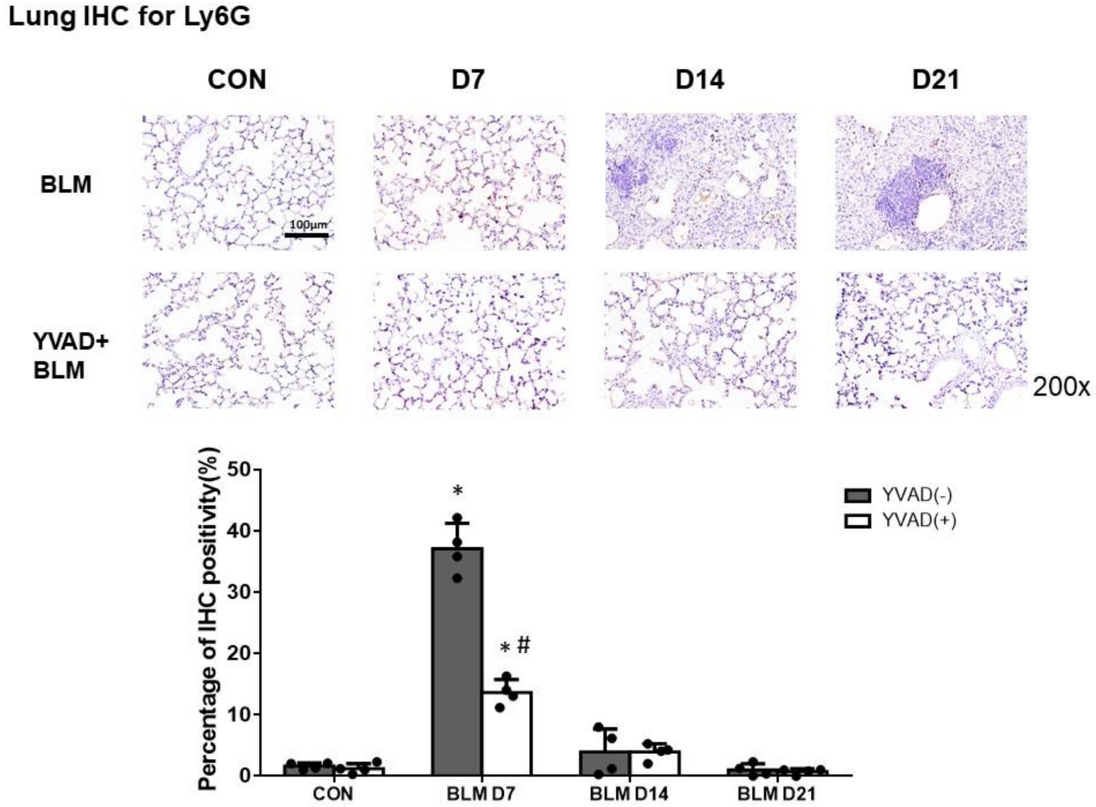

Supplementary Figure S1. Inhibition of NLRP3 inflammasome decreases neutrophil infiltration in mice with BLM-induced pulmonary fibrosis. IHC analysis shows that the administration of bleomycin increases Ly6G expression in lung tissues seven days after bleomycin injection, but YVAD decreases Ly6G expression. *$P < 0.05$ compared to the control, # $P < 0.05$ compared to the BLM group. n = 4 per group.
